# Co-citation analysis of molecular imaging in neuropsychiatric disorders: integrating perspectives from radiology, neuroscience, and psychiatry

**DOI:** 10.1093/psyrad/kkaf026

**Published:** 2025-09-23

**Authors:** Antonio Navarro-Ballester

**Affiliations:** Department of Radiology, Hospital General Universitario de Castellón, 12004 Castellón, Spain

**Keywords:** co-citation analysis, molecular imaging, neuropsychiatric disorders, bibliometrics, neuroimaging, radiology, VOSviewer

## Abstract

**Background:**

Molecular imaging plays a key role in advancing understanding of neuropsychiatric disorders. However, the conceptual structure of this interdisciplinary field remains poorly mapped from a bibliometric perspective. The objective of this study was to explore the intellectual structure and thematic development of research on molecular imaging applied to neuropsychiatric disorders using co-citation network analysis.

**Methods:**

A bibliometric co-citation analysis was conducted using data retrieved from Scopus. A targeted search strategy identified articles from 2014 to 2023 focused on MRS, fMRI, PET, and SPECT in the context of neuropsychiatric disorders. Bibliographic data were exported, and cited references were analyzed using VOSviewer. A manually curated thesaurus was applied to unify variant citations and reduce duplication. Co-citation networks were generated, and thematic clusters were identified and interpreted based on total link strength and citation density.

**Results:**

The co-citation network included 51 documents and revealed six major thematic clusters encompassing automated anatomical labeling and brain segmentation, functional and structural connectivity, affective neuroscience, clinical biomarkers, and methodological standardization. Notable references included foundational works on resting-state functional connectivity, motion correction, and diagnostic criteria for neuropsychiatric disorders. The clustering structure highlighted the convergence of radiology, neuroscience, and psychiatry around shared methodological tools and conceptual frameworks.

**Conclusion:**

Co-citation analysis revealed a well-defined and maturing intellectual landscape in molecular imaging applied to neuropsychiatry. The identified clusters represent distinct yet interconnected research lines, reflecting methodological innovation and translational potential. These findings offer a roadmap for future research, emphasizing methodological rigor, interdisciplinary collaboration, and clinical applicability.

## Introduction

Research in neuropsychiatry has undergone a transformation in recent decades with the incorporation of molecular imaging techniques, which allow for the exploration of brain activity beyond traditional structural findings. Modalities such as functional magnetic resonance imaging (fMRI), magnetic resonance spectroscopy (MRS), and positron emission tomography (PET) offer new opportunities to characterize the pathophysiological processes underlying mental disorders. In the absence of accessible non-invasive biomarkers, these techniques represent a unique approach to identifying neurochemical, metabolic, and functional alterations in the human brain (Zhan and Yu, 2015). For example, task-based fMRI has enabled the detection of dysfunctions in specific networks such as the default mode network or the central executive network in diseases such as schizophrenia or major depression (Marsman *et al*., 2013; Nord *et al*., 2021). MRS has made it possible to evaluate *in vivo* the brain concentrations of key metabolites such as glutamate, GABA, or *N*-acetylaspartate, levels of which have been associated with clinical symptoms and treatment response (Meyer *et al*., 2006; Marsman *et al*., 2013). Likewise, PET has been essential in studying dopaminergic neurotransmission in psychotic disorders and in characterizing inflammatory processes through the use of tracers such as [^11^C]-PK11195 (Turkheimer *et al*., 2007). These tools have made it possible, for the first time, to observe functional molecular alterations *in vivo*, thus providing an objective foundation for the study of disrupted brain circuits in mental illness.

Clinical interest in the use of molecular imaging in psychiatry has increased significantly in recent years, driven by the search for biomarkers that allow for more precise classification of disorders, prediction of therapeutic response, and the development of personalized interventions. Multimodal research—combining different imaging techniques—is emerging as a promising strategy to address the complexity of neuropsychiatric disorders. However, despite a growing body of literature, clinical translation remains limited due to methodological variability, high costs, and lack of standardized interpretation (Arbabshirani *et al*., 2017; Gong *et al*., 2019; Qi *et al*., 2020).

In this context, a detailed bibliometric analysis can provide a comprehensive view of the development of research on molecular imaging applied to neuropsychiatric disorders. Citation analysis, by quantifying how frequently a document is cited by others, enables the assessment of the influence and recognition of publications, authors, institutions, or countries within a given field. This approach also facilitates the temporal and geographic characterization of scientific output. On the other hand, co-citation analysis is based on the premise that two works jointly cited by a third party share some degree of thematic or conceptual relationship. By constructing co-citation networks, it becomes possible to identify thematic clusters and intellectual structures within the field, revealing established lines of research, links between scientific communities, and emerging areas that are not yet fully developed.

Despite the growing relevance of molecular imaging in psychiatry, few bibliometric studies have systematically explored this field using co-citation analysis. Previous works have either focused on single imaging modalities, such as PET in neuropsychiatric disorders (Cervenka *et al*., 2022) or fMRI in specific conditions such as attention deficit hyperactivity disorder (ADHD) (Zhu *et al*., 2008), or have not employed co-citation methods to map the intellectual structure of the literature. To our knowledge, no prior study has comprehensively examined the interdisciplinary domain of molecular neuroimaging across radiology, neuroscience, and psychiatry using a co-citation approach. The present study fills this gap by including all major molecular imaging techniques [fMRI, MRS, PET, and single-photon emission computed tomography (SPECT)] over a 10-year period (2014–2023), thereby capturing the broader thematic landscape of this evolving field.

The aim of this study was to conduct a co-citation analysis of the published literature on molecular imaging in neuropsychiatric disorders. This strategy enabled the identification of the most active research areas, main scientific actors, and emerging trends in a critical domain for the evolution of psychiatry toward a precision medicine model.

## Materials and methods

### Study design

An observational bibliometric study with a retrospective and exploratory approach was conducted, focused on structural analysis of the scientific literature on molecular imaging applied to neuropsychiatric disorders. The study was based on co-citation network analysis to identify the main intellectual currents and thematic areas in the field.

The methodological design followed the five-step workflow for science mapping studies using bibliometric techniques (Zupic and Cater, 2015). This approach, widely accepted in the specialized literature, includes: (i) defining the research questions and selecting the most appropriate bibliometric methods; (ii) systematically collecting data from a standardized bibliographic database; (iii) conducting quantitative analysis using citation and co-citation metrics; (iv) visualizing the resulting networks using specialized tools; and (v) interpreting the results to identify thematic clusters, consolidated intellectual structures, and knowledge gaps. These stages are described in detail in the following sections.

### Phase 1: definition of the bibliometric approach

The first phase of the study involved defining the research questions and selecting the most appropriate bibliometric methods to address them. Co-citation analysis was selected to obtain a structural characterization of the field of molecular imaging in neuropsychiatry. This approach allows for the exploration of underlying conceptual structures through co-citation patterns.

Based on this general objective, the following research questions were formulated:

- What intellectual structures and thematic lines can be identified through co-citation analysis?
- Are there knowledge gaps or emerging lines that may guide future clinical or translational research?

### Phase 2: data acquisition

Data extraction was performed on 5 April 5 2024 from the Scopus database (Elsevier), selected for its broad multidisciplinary coverage and its ability to export complete metadata, including cited references. In addition, Scopus offers unique author identifiers (Author ID), which reduce ambiguity in citation counts and enhance the accuracy of author-level metrics. This database has been used in similar bibliometric studies on neuroimaging and psychiatry for its reliability and compatibility with analytical tools such as VOSviewer (Gong *et al*., 2019; Canul-Medina *et al*., 2024; Zhang *et al*., 2024).

A search strategy was designed to retrieve relevant literature on molecular imaging in neuropsychiatric disorders. The following keywords were used in the article title: “MRS,” “fMRI,” “PET,” and “SPECT,” combined with “neuropsychiatric disorders” or “psychiatric disorders.” The search period was limited to the last 10 years (2014–2023) to capture the most current trends.

The search was restricted to documents published in journals classified under the following official Scopus subject areas, selected for their interdisciplinary relevance:

- Medicine: Radiology, Nuclear Medicine and Imaging; Psychiatry and Mental Health.
- Neuroscience: General Neuroscience; Cognitive Neuroscience.

Original articles, reviews, short communications, and letters to the editor were included. No language restrictions were applied. Conference proceedings, editorials without references, technical notes without bibliographic value, and documents without exportable metadata were excluded.

### Phase 3: data processing and co-citation analysis

Inconsistencies and duplicates were manually cleaned. The software VOSviewer (v.1.6.19) was then used to perform a co-citation analysis of cited documents. This technique enabled the identification of groups of frequently co-cited works, revealing the main intellectual currents, thematic areas, and structural relationships within the field of molecular imaging in neuropsychiatric disorders.

A co-citation matrix was generated based on the references cited by the included documents. To unify bibliographic variants of the same source, a manually created thesaurus file in tabulated format was applied, allowing the normalization of different citation forms of key books and articles. This cleaning step prevented duplications and improved the coherence of the resulting map.

A minimum threshold of 10 co-citations was initially applied to explore the intellectual structure of the field. This value was selected to balance comprehensiveness with interpretability. During optimization, the threshold was increased to 18 to improve cluster coherence and reduce peripheral noise. The networks were represented using VOSviewer’s attraction–repulsion layout algorithm. Thematic clusters were identified based on co-citation density and interpreted as potential intellectual structures of the field. Centrality, density, and connectivity metrics were evaluated to identify key nodes within each cluster.

To validate the robustness and consistency of the network, the data were exported to Gephi (v.0.10.1), where the visualizations were replicated using different graphic parameters.

The 2014–2023 time window was selected to capture the most recent decade of research, a period characterized by major methodological advances (resting-state fMRI, PET neuroinflammation tracers, multimodal fusion approaches) and a progressive incorporation of imaging biomarkers into psychiatric research. This ensured that the analysis reflected contemporary and clinically relevant trends, while avoiding the heterogeneity of older studies with outdated techniques. The adjustment from 10 to 18 co-citations was guided by exploratory testing, which showed that this higher threshold improved the cohesion and interpretability of the clusters by reducing peripheral noise while preserving the main intellectual communities. This optimization process, consistent with best practices in bibliometric mapping, allowed the network to remain dense enough for analysis but sufficiently refined to identify meaningful clusters.

### Phase 4: interpretation of results

The final phase of the methodological workflow consisted of qualitative interpretation of the results obtained from the co-citation analysis. This interpretation focused on two main dimensions:

1. Intellectual structure: the clusters generated in the co-citation analysis were examined to identify coherent thematic groupings, consolidated lines of research, and relevant conceptual connections.
2. Implications for future research: thematic gaps and areas with lower co-citation density were evaluated as potential opportunities for new studies. The translational potential of the identified clusters was also considered in relation to the development of clinically applicable biomarkers in psychiatry.

The interpretation was supported by cross-referencing the works included in each cluster and by the contextual analysis of their main contributions, avoiding automatic inferences based solely on quantitative metrics. The findings are discussed in detail in the Results and Discussion sections.

### Ethical considerations

As this study relied exclusively on publicly available bibliographic data, approval by an ethics committee and informed consent were not required.

## Results

A total of 245 486 cited references were identified in the included documents. For the co-citation analysis, a minimum threshold of 18 co-citations was established, allowing the inclusion of references with significant and recurring presence in the field’s literature. This value was selected after exploratory testing with a lower threshold (*n* = 10), which produced an overly dense and fragmented network. The final threshold enhanced interpretability and reduced peripheral noise while retaining the core intellectual structure. Although 52 documents surpassed this threshold, only 51 were included in the final network, as one was not connected to the main component and was excluded from the visualized map.

**Figure 1:**
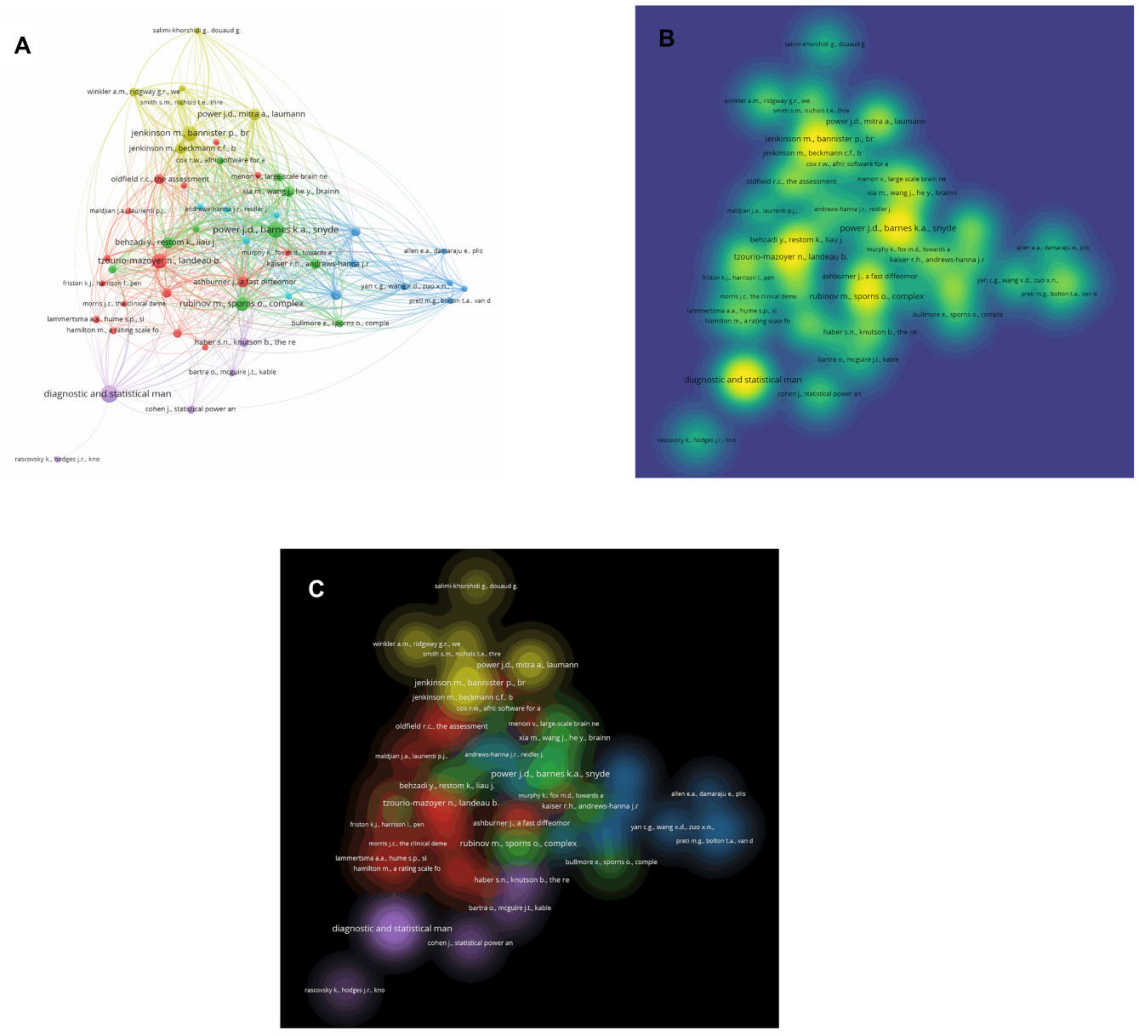
Co-citation network visualization. (**A**) Network view with clusters identified by color. Each node represents a highly co-cited document; edge thickness indicates co-citation strength. (**B**) Density visualization highlighting areas of thematic concentration; warmer colors indicate a higher concentration of co-citations. (**C**) Cluster density visualization emphasizing the conceptual weight and overlap between co-citation communities.

The resulting network was visualized using VOSviewer software (v.1.6.19), applying its attraction–repulsion layout algorithm. Each node represents a cited reference, and the colors indicate the thematic clusters automatically detected. A map composed of six clusters was obtained, interpreted as conceptual groupings around different lines of research within the field of molecular imaging in neuropsychiatric disorders (Fig. 1A). The density visualization highlights the frequency of co-citations across the network (Fig. 1B), while the clustered representation illustrates the thematic communities and their structural relationships (Fig. 1C).

The co-citation analysis revealed a structure composed of six distinct thematic clusters (Table 1).

**Table 1:** Most co-cited documents by community. The three documents with the highest Weight (corresponding to Total link strength in VOSviewer) in each of the six communities shown in Fig. 1 are presented. Each color in the table corresponds to the color of the community in the co-citation network visualization.

Community		Topic (key words)	Reference	Total link strength	Citations
**1**	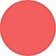	Algorithms, Artificial Intelligence, Brain Mapping/methods, Image Enhancement/methods, Image Interpretation, Memory/physiology	Tzourio-Mazoyer N., 2002, *Neuroimage*, 15, p273, doi: 10.1006/nimg.2001.0978	123	105
			Ashburner J., 2007, *Neuroimage*, 38, p95, doi: 10.1016/j.neuroimage.2007.07.007	123	58
			Ashburner J., 2005, *Neuroimage*, 26, p839, doi: 10.1016/j.neuroimage.2005.02.018	67	41
**2**	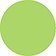	Brain/anatomy & histology, Brain/physiology, Image Processing, Computer-Assisted, Magnetic Resonance Imaging	Power J.D., 2012, *Neuroimage*, 59, p2142, doi: 10.1016/j.neuroimage.2011.10.018	238	124
			Rubinov M., 2010, *Neuroimage*, 52, p1059, doi: 10.1016/j.neuroimage.2009.10.003	147	92
			Behzadi Y., 2007, *Neuroimage*, 37, p90, doi: 10.1016/j.neuroimage.2007.07.007	112	64
**3**	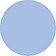	Adolescent, Adult, Animals, Child, Humans, Female, Fingers/physiology, Hand/physiology, Male, Time Factors	Biswal B., 1995, *Magn Reson Med*, 34, p537, doi: 10.1002/mrm.1 910 340 409	125	49
			Fox M.D., 2007, *Nat Rev Neurosci*, 8, p700, doi: 10.1038/nrn2201	106	41
			Yan C.G., 2016, *Neuroinformatics*, 14, p339, doi: 10.1007/s12021-016-9299–4	65	26
**4**	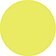	Acoustic Stimulation, Brain Mapping/methods, Models, Neurological, Motion, Photic Stimulation, Reproducibility of Results, Software/history	Jenkinson M., 2002, *Neuroimage*, 17, p825, doi: 10.1016/s1053-8119(02)91132–8	179	102
			Power J.D., 2014, *Neuroimage*, 84, p320, doi: 10.1016/j.neuroimage.2013.08.048	126	60
			Jenkinson M., 2012, *Neuroimage*, 62, p790, doi: 10.1016/j.neuroimage.2011.09.015	106	55
**5**	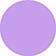	Choice Behavior/physiology, Diagnostic and Statistical Manual of Mental Disorders, International Classification of Diseases, Psychotic Disorders/diagnosis	American Psychiatric Association, 2013, American Psychiatric Publishing, p1, doi: 10.1176/appi.books.9780890425596	55	140
			Haber S.N., 2010, *Neuropsychopharmacology*, 35, p4, doi: 10.1038/npp.2009.129	30	41
			Bartra O., 2013, *Neuroimage*, 76, p412, doi: 10.1016/j.neuroimage.2013.02.063	20	22
**6**	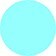	Biological Clocks/physiology, Cluster Analysis, Cognition, Computer Simulation, Emotions/physiology, Models, Nerve Net/physiology, Statistics as Topic	Murphy K., 2009, *Neuroimage*, 44, p893, doi: 10.1016/j.neuroimage.2008.09.036	64	23
			Seeley W.W., 2007, *J Neurosci*, 27, p2349, doi: 10.1523/JNEUROSCI.5587–06.2007	54	20
			Seeley W.W., 2009, *Neuron*, 62, p42, doi: 10.1016/j.neuron.2009.03.024	52	19

### Cluster 1. Anatomical segmentation and image processing

This cluster was dominated by references on anatomical labeling and segmentation methods. Tzourio-Mazoyer *et al*. (2002) introduced the widely used Automated Anatomical Labeling (AAL) atlas, while Ashburner (2007) developed diffeomorphic registration methods. These works provided the structural framework that underpins many functional and structural imaging analyses.

### Cluster 2. Structural and functional connectivity

Connectivity research was represented by methodological studies such as Power *et al*. (2012), which highlighted the influence of motion artifacts, and Rubinov and Sporns (2010), who formalized graph-theoretical metrics for brain networks. Together with Behzadi *et al*. (2007) on physiological noise correction, these works established standards for reliable connectivity mapping.

### Cluster 3. Resting-state functional connectivity in clinical populations

This cluster included seminal studies such as Biswal *et al*. (1995), who first demonstrated resting-state functional connectivity, and Fox and Raichle (2007), who expanded its clinical relevance. Subsequent works (e.g. Yan *et al*., 2016) developed computational tools to analyze resting-state networks in psychiatric cohorts, consolidating their use as biomarkers of altered network organization.

### Cluster 4. Acquisition methods and standardization

Cluster 4 grouped methodological contributions essential for study reproducibility. Jenkinson *et al*. (2002) and Jenkinson *et al*. (2012) advanced robust motion correction and registration tools, while Power *et al*. (2014) refined artifact detection in fMRI. These studies provided technical solutions that are critical for data quality and comparability across sites.

### Cluster 5. Psychiatric diagnostic frameworks and neurobiology

This cluster contained references linking imaging to diagnostic systems. The DSM-5 (American Psychiatric Association, 2013) defined standardized psychiatric criteria, while Haber and Knutson (2009) and Bartra *et al*. (2013) integrated imaging findings into models of reward and decision-making. These works exemplify the bridge between neurobiological evidence and clinical classification.

### Cluster 6. Brain dynamics and affective processes

Cluster 6 incorporated literature on salience and cognitive-emotional networks. Seeley *et al*. (2007, 2009) described intrinsic connectivity systems underlying social and affective processing, while Murphy *et al*. (2009) proposed analytical models for dynamic brain states. This community reflects the integration of cognitive and affective neuroscience within imaging research.

### Interconnections between clusters

The map also revealed cross-links between communities. Methodological clusters (1 and 4) were tightly connected with connectivity research (2), while clinical application clusters (3 and 5) linked with affective neuroscience (6). These relationships indicate how technical advances, connectivity measures, and psychiatric frameworks converge in the field.

PET-related references are represented among the most co-cited sources, including molecular imaging studies in depression and neuroinflammation (e.g. Meyer *et al*., 2006; Turkheimer *et al*., 2007; Cervenka *et al*., 2022). Although SPECT was included in the scope of the search, no SPECT-focused references reached the co-citation threshold or connected to the main component, which explains their absence from the network.

## Discussion

The co-citation analysis allowed the identification of six thematic clusters that represent different methodological and conceptual streams in the use of molecular imaging for the study of neuropsychiatric disorders. This organization not only reflects bibliographic groupings but also demonstrates how disciplines such as radiology, neuroscience, and psychiatry converge around shared objectives.

Some clusters stand out for their strong technical and methodological focus. Cluster 1, centered on automated anatomical segmentation tools and neuroimaging processing, highlights the essential role of radiology in developing analytical methods to obtain brain biomarkers. These tools constitute the technical foundation upon which many clinical and research applications are based. A notable example within this cluster is the work by Tzourio-Mazoyer *et al*. (2002), which introduced the AAL atlas as a key reference for anatomical segmentation in neuroimaging studies. Its high co-citation frequency reflects its broad methodological impact and its adoption as a standard in many functional and structural analyses.

**Figure 2:**
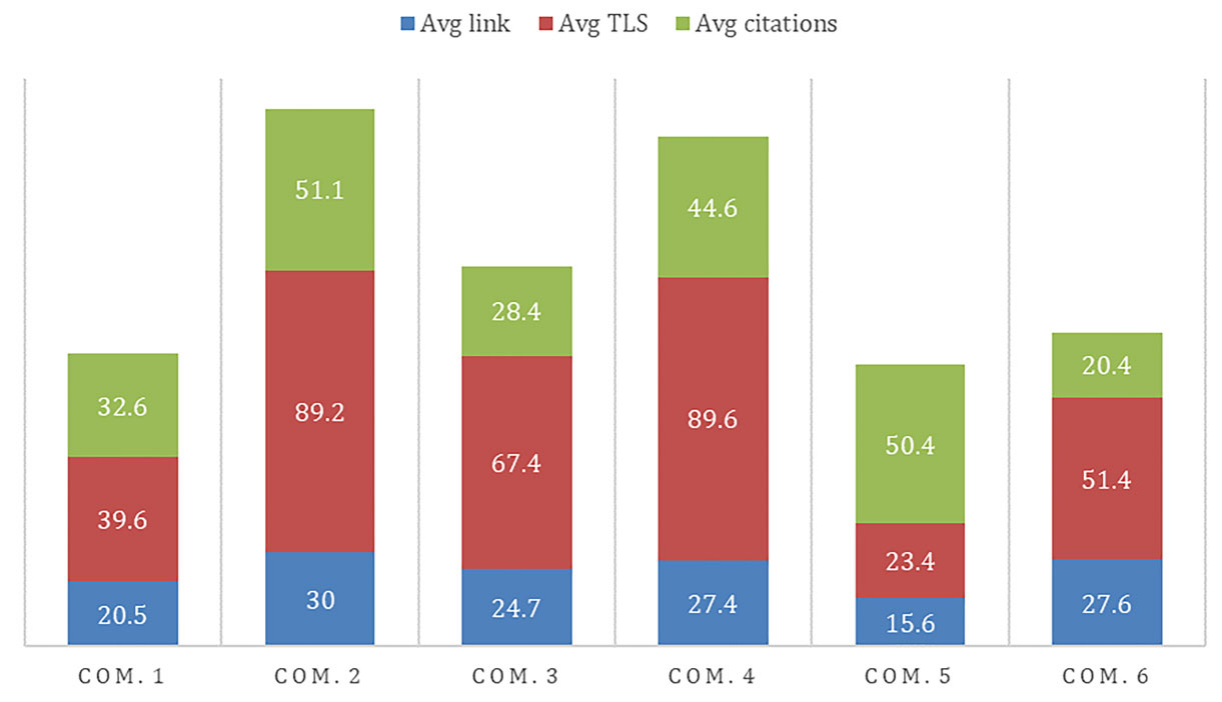
Average bibliometric metrics by co-citation community. The chart shows the average (Avg) number of links, total link strength (TLS), and citations for the documents in each co-citation community (COM.). Blue bars represent average links, red bars indicate average co-citation weight (TLS), and green bars correspond to average citations. These values reflect the structural cohesion, conceptual centrality, and scholarly impact of each thematic group.

Other groups, such as Clusters 2 and 6, combine methodological and conceptual aspects. Cluster 2 integrates studies on structural and functional brain connectivity along with advanced network analyses, positioning itself in a space shared between computational neuroscience and quantitative radiology. This community stands out for showing the highest average Total link strength, suggesting strong conceptual centrality in the co-citation network (Fig. 2). The most representative document in this group is the work by Power *et al*. (2012), which highlighted how subject movement can introduce spurious but systematic correlations in functional connectivity analyses. This finding had a major methodological impact, prompting the development of new motion correction strategies and redefining standards in functional neuroimaging analysis.

Cluster 6, on the other hand, focuses on brain dynamics and functional networks involved in cognitive and emotional processes, reflecting the interest of affective and cognitive neuroscience in understanding higher brain functions. This thematic community includes studies on the interaction between emotion, cognition, and brain systems. It aligns with affective neuroscience, which explores the neural mechanisms behind motivation, effect, and decision-making. This line of research is also connected with the development of neuroimaging tools applied to affective dimensions such as emotional reactivity, regulation, and social processing—areas of increasing clinical relevance in the study of disorders such as depression, anxiety, and conduct disorder (Penner *et al*., 2018).

Cluster 3 stands out for its higher average number of citations, indicating particularly strong academic impact, possibly linked to its clinical applicability in populations with neuropsychiatric disorders (Fig. 2). The link with psychiatry is clearly evidenced in Cluster 3, which analyzes resting-state functional connectivity in clinical populations, and in Cluster 5, which gathers diagnostic references such as the Diagnostic and Statistical Manual of Mental Disorders (DSM-5) and psychometric scales. These groups represent a direct connection between neuroimaging findings and clinical practice aimed at characterizing mental disorders. For example, the study by Biswal *et al*. (1995), included in Cluster 3, introduced the concept of resting-state functional connectivity and marked the beginning of a research line that later expanded into psychiatric pathologies. Similarly, the article by Rascovsky *et al*. (2011) in Cluster 5 stands out for its contribution to redefining the diagnostic criteria of frontotemporal dementia, integrating clinical correlates and neurobiological findings. The presence of these references reflects how functional neuroimaging has become a bridge between basic research and clinical evaluation in psychiatry.

Cluster 4 shows lower averages in both links and citations, suggesting a more specialized role within the thematic network, focused on technical issues essential to study validity. It gathers works on image acquisition, motion artifact correction, and data standardization—areas that are essential to ensure reproducibility and comparability in neuroimaging. In a context of growing attention to the reproducibility crisis in neuroscience, this cluster acquires strategic relevance: methodological standardization and analytical transparency have been identified as pillars to generate solid, replicable, and generalizable results (Poldrack, 2019; Botvinik-Nezer and Wager, 2023).

This co-citation network-based approach has been successfully used in other bibliometric studies applied to the field of neuroimaging. For example, Deng *et al*. (2023) identified thematic clusters related to functional connectivity, neurofeedback, and ADHD, confirming the utility of this method for mapping scientific communities in highly specialized fields.

Similarly, co-citation analyses have been used to examine the scientific response to emerging challenges in mental health, such as during the COVID-19 pandemic (Chen *et al*., 2021; Navarro-Ballester *et al*., 2023). Building on these efforts, our study applies this methodology to the domain of molecular imaging in psychiatry, revealing a more mature and interdisciplinary thematic structure. Previous bibliometric works have focused on individual imaging techniques, including PET in neuropsychiatric disorders (Cervenka *et al*., 2022), fMRI in ADHD (Zhu *et al*., 2008), and mood disorders through keyword analysis (Gao *et al*., 2021; Lu *et al*., 2022), or have provided narrative overviews of hybrid imaging approaches (Burhan *et al*., 2015). Our co-citation map extends these findings by demonstrating that PET-related works are embedded in a broader multimodal context together with fMRI and MRS, while MRI-based connectivity studies co-occur with psychiatric diagnostic frameworks (Cluster 5). This indicates that the field has moved from modality-specific analyses toward interdisciplinary clusters that link imaging methods with clinical translation.

The interdisciplinary nature of the field is also visible in the composition of the clusters. Methodological communities (Clusters 1 and 4) are primarily anchored in radiology and imaging journals, while Clusters 3 and 5 reflect psychiatry-oriented frameworks, and Cluster 6 draws heavily from affective neuroscience. Importantly, certain clusters bridge disciplinary boundaries: connectivity studies (Cluster 2) link methodological advances with clinical research in psychiatric populations (Cluster 3), and diagnostic frameworks (Cluster 5) co-occur with imaging-based clusters (3 and 6). Journals such as *Neuroimage* and *Biological Psychiatry*, and research groups working across imaging and psychiatry, appear in multiple clusters, illustrating the integrative role of these outlets and communities. These patterns provide concrete evidence that the co-citation network does not reproduce disciplinary silos but highlights genuine cross-talk between radiology, neuroscience, and psychiatry.

The absence of SPECT among the most co-cited references reflects the network inclusion criteria rather than an a priori exclusion, probably due to lower co-citation frequency compared with PET during the study period. The identification of six well-defined clusters points to an evolving thematic landscape that can inform future translational efforts.

Overall, the co-citation network not only reveals consolidated research lines but also helps identify thematic gaps. Some clusters exhibit low connectivity or sparse density, suggesting areas of knowledge that are less developed or lack conceptual integration. This finding underscores the need to promote research that bridges still-fragmented domains, such as the intersection between molecular neuroimaging and complex affective disorders, or the development of clinically validated translational biomarkers.

Beyond their individual content, the clusters also reveal significant cross-links that highlight the interdisciplinary nature of the field. Methodological clusters (1 and 4), which focus on segmentation and data standardization, show strong connections with connectivity studies (2), underlining how technical advances directly support the reliability of functional and structural analyses. Likewise, the linkage between connectivity clusters (2 and 3) and affective neuroscience (6) points to a convergence of cognitive and emotional domains that are highly relevant in psychiatric research. Finally, the overlap between clinical diagnostic frameworks (5) and imaging-based clusters (3 and 6) illustrates ongoing efforts to align neurobiological findings with psychiatric classification systems. Together, these interconnections emphasize that progress in molecular neuroimaging depends on the integration of methodological innovation, conceptual neuroscience, and clinical translation.

### From clusters to clinics

The thematic clusters identified through our co-citation analysis not only map the intellectual structure of the field, but also suggest specific avenues for clinical translation. Cluster 3, for instance, centers around studies using resting-state fMRI and connectivity metrics to identify biomarkers of altered brain network organization in psychiatric populations. The consolidation of this domain indicates growing interest in using imaging-derived functional markers to stratify patient subtypes, predict treatment response, or monitor disease progression. These developments are particularly relevant for disorders with heterogeneous clinical presentations, such as depression or schizophrenia.

Cluster 5, by contrast, encompasses literature related to diagnostic classification systems (e.g. DSM-5), symptom-based subtyping, and the integration of imaging findings into nosological frameworks. The presence of this cluster suggests increasing efforts to anchor psychiatric diagnosis in neurobiological substrates, potentially informing the transition toward dimensional or circuit-based models of mental illness. This line of work may contribute to the refinement of diagnostic criteria, the validation of imaging-guided treatment algorithms, and the development of precision psychiatry protocols.

Together, these clusters exemplify how bibliometric mapping can highlight not only scientific activity but also translational momentum. By identifying well-structured research domains with high citation coherence and clinical relevance, co-citation analysis may help prioritize areas for future investment and interdisciplinary collaboration.

These observations reinforce the potential of co-citation analysis not only as a mapping tool but also as a guide for identifying translational priorities in psychiatric neuroimaging.

### Limitations

This study has several limitations. First, the analysis was based exclusively on the Scopus database. While Scopus offers broad multidisciplinary coverage and compatibility with VOSviewer, the exclusion of other databases such as Web of Science may have led to the omission of relevant publications. Second, the search was restricted to article titles, which might have excluded pertinent studies that mention key concepts only in abstracts or keywords. Third, preprints, conference abstracts, and non-English language publications were not included, as non-English papers did not reach the citation threshold required for inclusion in the co-citation network, potentially introducing a publication and language bias. Lastly, although co-citation analysis provides a robust overview of the intellectual structure of a field, it does not capture recent developments that have not yet accumulated sufficient citations.

## Conclusion

This study demonstrates that co-citation analysis is an effective tool for identifying intellectual structures and scientific communities in the field of molecular imaging applied to neuropsychiatric disorders. The six identified clusters reflect a progressive integration of radiology, neuroscience, and psychiatry, as well as a growing interest in methodological standardization, clinical applicability, and translational research.

Beyond mapping the thematic landscape, our findings highlight specific translational opportunities. Cluster 3, focused on functional connectivity, points toward the development of neuroimaging-based biomarkers to support differential diagnosis and personalized treatment strategies. Cluster 5 emphasizes the integration of neuroimaging findings into psychiatric classification systems, opening avenues for diagnostic refinement and early detection. These examples underscore how bibliometric insights can help prioritize clinical applications and guide multidisciplinary collaborations.

Future investigations should aim to strengthen the connection between technical innovation and patient-centered outcomes by validating imaging protocols in real-world clinical settings and promoting the development of evidence-based translational tools.

## Data sharing statement

All data generated or analyzed during the study are included in the published paper.

## References

[doi35_189_050625] American Psychiatric Association (2013) Diagnostic and Statistical Manual of Mental Disorders, 5th edn. Arlington, VA: American Psychiatric Publishing.

[bib1] Arbabshirani MR, Plis S, Sui J et al. (2017) Single subject prediction of brain disorders in neuroimaging: promises and pitfalls. Neuroimage. 145:137–65. 27012503 10.1016/j.neuroimage.2016.02.079 PMC5031516

[bib30_620_054825] Ashburner J (2007) A fast diffeomorphic image registration algorithm. Neuroimage. 38:95–113. 17761438 10.1016/j.neuroimage.2007.07.007

[doi37_838_051125] Bartra O, McGuire JT, Kable JW (2013) The valuation system: a coordinate-based meta-analysis of BOLD fMRI experiments examining neural correlates of subjective value. NeuroImage. 76:412–27. 23507394 10.1016/j.neuroimage.2013.02.063 PMC3756836

[bib2] Behzadi Y, Restom K, Liau J et al. (2007) A component based noise correction method (CompCor) for BOLD and perfusion based fMRI. Neuroimage. 37:90–101. 17560126 10.1016/j.neuroimage.2007.04.042 PMC2214855

[bib3] Biswal B, Zerrin Yetkin F, Haughton VM et al. (1995) Functional connectivity in the motor cortex of resting human brain using echo-planar MRI. Magnetic Resonance Med. 34:537–41. 10.1002/mrm.1910340409 8524021

[bib4] Botvinik-Nezer R, Wager TD (2023) Reproducibility in neuroimaging analysis: challenges and solutions. Biol Psychiatry Cogn Neurosci Neuroim. 8:780–8. 10.1016/j.bpsc.2022.12.006 36906444

[bib5] Burhan A, Marlatt N, Palaniyappan L et al. (2015) Role of hybrid brain imaging in neuropsychiatric disorders. Diagnostics. 5:577–614. 26854172 10.3390/diagnostics5040577 PMC4728476

[bib6] Canul-Medina G, López-Pech G, Jiménez-Trejo F (2024) Global research in schizophrenia and serotonin: a bibliometric analysis. Front Psychiatry. 15:1436906. 39156608 10.3389/fpsyt.2024.1436906 PMC11329940

[bib7] Cervenka S, Frick A, Bodén R et al. (2022) Application of positron emission tomography in psychiatry: methodological developments and future directions. Transl Psychiatry. 12:248. 35701411 10.1038/s41398-022-01990-2 PMC9198063

[bib8] Chen Y, Zhang X, Chen S et al. (2021) Bibliometric analysis of mental health during the COVID-19 pandemic. Asian J Psychiatry. 65:102846. 10.1016/j.ajp.2021.102846 PMC8435062 34562753

[bib9] Deng H, Huang Z, Li Z et al. (2023) Systematic bibliometric and visualized analysis of research hotspots and trends in attention-deficit hyperactivity disorder neuroimaging. Front Neurosci. 17:1098526. 37056309 10.3389/fnins.2023.1098526 PMC10086162

[bib31_551_055125] Fox MD and Raichle ME (2007) Spontaneous fluctuations in brain activity observed with functional magnetic resonance imaging. Nat Rev Neurosci. 8:700–11. 17704812 10.1038/nrn2201

[bib10] Gao M, Sun H, Cheng X et al. (2021) Magnetic resonance imaging in mood disorders: a bibliometric analysis from 1999 to 2020. Clin Transl Imaging. 9:241–54.

[bib11] Gong B, Naveed S, Hafeez DM et al. (2019) Neuroimaging in psychiatric disorders: a bibliometric analysis of the 100 most highly cited articles. J Neuroimaging. 29:14–33. 30311320 10.1111/jon.12570

[bib12] Gong Q, Lui S, Sweeney JA (2019) A selective review of cerebral abnormalities in patients with first-episode schizophrenia before and after treatment. Biol Psychiatry. 85:281–91. 26621570 10.1176/appi.ajp.2015.15050641

[doi36_786_050825] Haber SN, Knutson B (2009) The reward circuit: linking primate anatomy and human imaging. Neuropsychopharmacology. 35:4–26. 10.1038/npp.2009.129 PMC3055449 19812543

[bib33_162_055525] Jenkinson M, Bannister P, Brady M et al. (2002) Improved optimization for the robust and accurate linear registration and motion correction of brain images. Neuroimage. 17:825–41. 12377157 10.1016/s1053-8119(02)91132-8

[bib34_148_050125] Jenkinson M, Beckmann CF, Behrens TE et al. (2012) FSL. Neuroimage. 62:782–90. 21979382 10.1016/j.neuroimage.2011.09.015

[bib13] Lu Y, Zhang L, Wu X-Y et al. (2022) Systematic bibliometric and visualized analysis of research hotspots and trends on autism spectrum disorder neuroimaging. Dis Markers. 2022:1. 10.1155/2022/3372217 PMC9313970 35899177

[bib14] Marsman A, van den Heuvel MP, Klomp DWJ et al. (2013) Glutamate in schizophrenia: a focused review and meta-analysis of (1)H-MRS studies. Schizophr Bull. 39:120–9. 21746807 10.1093/schbul/sbr069 PMC3523901

[bib15] Meyer JH, Ginovart N, Boovariwala A et al. (2006) Elevated monoamine oxidase A levels in the brain: an explanation for the monoamine imbalance of major depression. Arch Gen Psychiatry. 63:1209–16. 17088501 10.1001/archpsyc.63.11.1209

[doi40_216_051625] Murphy K, Birn RM, Handwerker DA et al. (2009) The impact of global signal regression on resting state correlations: are anti-correlated networks introduced?. NeuroImage. 44:893–905. 18976716 10.1016/j.neuroimage.2008.09.036 PMC2750906

[bib16] Navarro-Ballester A, Merino-Bonilla JA, Ros-Mendoza LH et al. (2023) Publications on COVID-19 in radiology journals in 2020 and 2021: bibliometric citation and co-citation network analysis. Eur Radiol. 33(5):3103–14. 36571605 10.1007/s00330-022-09340-y PMC9791158

[bib17] Nord CL, Valton V, Wood J et al. (2021) Unreliability of putative fMRI biomarkers during emotional face processing. Neuroimage. 224:117413 28506872 10.1016/j.neuroimage.2017.05.024 PMC5553850

[bib18] Penner AE, Stoddard J (2018) Clinical affective neuroscience. J Am Acad Child Adolescent Psychiatry. 57:906–8. 10.1016/j.jaac.2018.07.877 PMC6607912 30522733

[bib19] Poldrack RA (2019) The costs of reproducibility. Neuron. 101:11–4. 30605654 10.1016/j.neuron.2018.11.030

[bib20] Power JD, Barnes KA, Snyder AZ et al. (2012) Spurious but systematic correlations in functional connectivity MRI networks arise from subject motion. Neuroimage. 59(3):2142–54. 22019881 10.1016/j.neuroimage.2011.10.018 PMC3254728

[bib41_163_304525] Power JD, Mitra A, Laumann TO et al. (2014) Methods to detect, characterize, and remove motion artifact in resting state fMRI. Neuroimage. 84:320–341. 23994314 10.1016/j.neuroimage.2013.08.048 PMC3849338

[bib21] Qi S, Calhoun VD, van Erp TGM et al. (2020) Multimodal fusion with reference: searching for joint neuromarkers of working memory deficits in schizophrenia. IEEE Trans Med Imaging. 39:2630–40. 10.1109/TMI.2017.2725306 PMC5750081 28708547

[bib22] Rascovsky K, Hodges JR, Knopman D et al. (2011) Sensitivity of revised diagnostic criteria for the behavioural variant of frontotemporal dementia. Brain. 134:2456–77. 21810890 10.1093/brain/awr179 PMC3170532

[bib23] Rubinov M, Sporns O (2010) Complex network measures of brain connectivity: uses and interpretations. Neuroimage. 52:1059–69. 19819337 10.1016/j.neuroimage.2009.10.003

[doi39_957_051425] Seeley WW, Crawford RK, Zhou J et al. (2009) Neurodegenerative diseases target large-scale human brain networks. Neuron. 62:42–52. 19376066 10.1016/j.neuron.2009.03.024 PMC2691647

[bib38_760_051325] Seeley WW, Menon V, Schatzberg AF et al. (2007) Dissociable intrinsic connectivity networks for salience processing and executive control. J Neurosci. 27:2349–56. 17329432 10.1523/JNEUROSCI.5587-06.2007 PMC2680293

[bib24] Turkheimer FE, Edison P, Pavese N et al. (2007) Reference and target region modeling of [11C]-(R)-PK11195 brain studies. J Nucl Med. 48:158–67. 17204713

[bib25] Tzourio-Mazoyer N, Landeau B, Papathanassiou D et al. (2002) Automated anatomical labeling of activations in SPM using a macroscopic anatomical parcellation of the MNI MRI single-subject brain. Neuroimage. 15:273–89. 11771995 10.1006/nimg.2001.0978

[bib32_339_055325] Yan CG, Wang XD, Zuo XN et al. (2016) DPABI: data processing & analysis for (resting-state) brain imaging. Neuroinformatics. 14:339–51. 27075850 10.1007/s12021-016-9299-4

[bib26] Zhan X, Yu R (2015) A window into the brain: advances in psychiatric fMRI. Biomed Res Int. 2015:1. 10.1155/2015/542467 PMC4564608 26413531

[bib27] Zhang P, Zhang J, Wang M et al. (2024) Research hotspots and trends of neuroimaging in social anxiety: a CiteSpace bibliometric analysis based on Web of Science and Scopus database. Front Behav Neurosci. 18:1448412. 39713279 10.3389/fnbeh.2024.1448412 PMC11659959

[bib29_497_054025] Zhu CZ, Zang YF, Cao QJ et al. (2008) Fisher discriminative analysis of resting-state brain function for attention-deficit/hyperactivity disorder. Neuroimage. 40:110–20. 18191584 10.1016/j.neuroimage.2007.11.029

[bib28] Zupic I, Čater Tž (2015) Bibliometric methods in management and organization. Organizational Res Methods. 18:429–72.

